# Virtual respiratory system in investigation of CPAP influence on optimal breathing frequency in obstructive lungs disease

**DOI:** 10.1186/1753-4631-1-6

**Published:** 2007-07-16

**Authors:** Tomasz Golczewski, Marek Darowski

**Affiliations:** 1 Institute of Biocybernetics and Biomedical Engineering, Polish Academy of Sciences, Warsaw, Poland

## Abstract

**Background:**

Continuous Positive Airway Pressure (CPAP) is a commonly accepted method of spontaneous breathing support in obstructive lung disease. Previous work suggested that the cause of the CPAP efficacy in the obstructive lung disease localized in bronchi of middle order (OLDMO) is not as obvious as, for example, in the obstructive sleep apnea. Since CPAP reduces obstruction and the optimal breathing frequency (BF) depends on the obstruction level, it seems to be important to analyze the dependence of the optimal BF on CPAP.

**Aim:**

To analyze the support efficacy cause in OLDMO, esp. the relationship between the CPAP value and optimal BF.

**Method:**

Investigations utilized previously built virtual respiratory system. Its most important factors: nonlinear lungs compliance and changeability of nonlinear airway resistance (Raw). Influence of BF and the CPAP value on the tidal volume and minute ventilation was analyzed for four exemplary virtual patients: healthy ("standard") and suffering from moderate, severe, and the very severe OLDMO (the other parameters, esp. respiratory muscles effort, were unchanged). Minute inspiratory work as a criterion of the BF optimization.

**Results:**

CPAP decreased Raw making breathing easier, however, it shifted the working point of the respiratory system towards the smaller lungs compliance making breathing harder. The final result depended on the Raw value: CPAP improved breathing of patients with the serious OLDMO while it worsened healthy person breathing. The optimal CPAP value depended on the Raw value. If a virtual patient suffering from the serious OLDMO was not supported with CPAP, he had to breathe with low frequency because minute ventilation did not rise with BF increase. The optimal BF depended on the CPAP value (the greater the value, the greater the frequency).

**Conclusion:**

The CPAP efficacy depends on the level of OLDMO. CPAP is efficient in the severe OLDMO because it increases the optimal BF, which makes possible less energy-consuming breathing with frequency close to the normal one (greater BF means smaller tidal volume and thus smaller work against lungs compliance).

## 1. Background

To avoid ethical and economic problems connected with experiments and investigations on animals or human beings (patients), the Virtual Respiratory System (VRS) has been recently developed in the Institute of Biocybernetics and Biomedical Engineering in Warsaw [1,2]. Testing and analyses of various artificial ventilation and ventilatory support methods were original applications of VRS [3,4].

The paper presents analysis of efficacy of spontaneous breathing support by means of the Continuous Positive Airway Pressure (**CPAP**) in patients suffering from obstructive lung disease. Despite one name, three types of such disease may be distinguished. The type depends on the obstruction localization. If the obstruction concerns the upper airway (*e.g*. as in the sleep apnea), the cause of the CPAP efficacy seems to be clear: CPAP keeps the upper airway open. Sullivan *et al *used CPAP in sleep apnea as the first [5] and nasal CPAP is a standard therapy for obstructive sleep apnea syndrome, now. If the obstruction that concerns smallest bronchi is connected with atelectasis, then the efficacy cause seems to be clear, too: CPAP prevents alveoli from collapse.

However, if the obstruction concerns the bronchi of the middle order (*e.g*. asthma), the efficacy cause is not so obvious. Although easier inspiration is usually supposed to be the efficacy cause, our previous investigation with VRS [2] has shown that the expiration period is much more critical in this case. It is because both the obstruction and transmural pressure (pressure inside bronchi minus intrapleural pressure) influence airflow through such bronchi: the smaller the transmural pressure because of increased intrapleural pressure during the expiration, the greater the summarized influence of the obstruction and the pressure on the airflow. Since intrapleural pressure rise depends on the duration of the expiration, and thus on the breathing frequency, the frequency has to be taken into account in an analysis of the CPAP efficacy.

Summarizing: this paper presents detailed analysis of the CPAP efficacy in the case of patients with obstruction localized in the bronchi of middle generations. The analysis concerns dependence of the tidal volume on breathing frequency, obstruction level, and CPAP, when force developed by respiratory muscles is constant.

## 2. Methods

### 2.1. Virtual respiratory system

**Figure 1:**
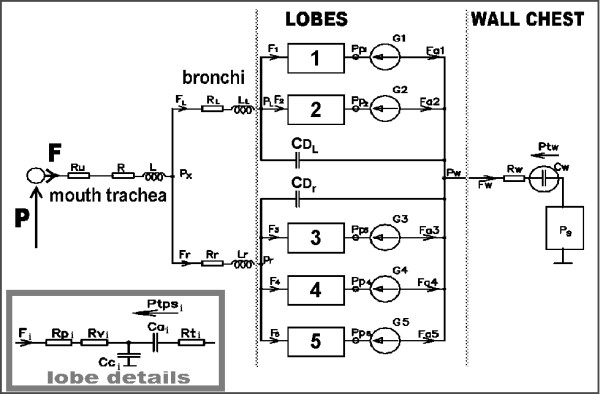
**Simplified scheme of the respiratory mechanics model**. Ru, R, R_L_, R_r _– upper airway resistances, L, L_L_, L_r _– inertances, C_DL_, C_Dr _– the parts of dead space different from the trachea and main bronchi (indices _L _and _r _concern the left and right lung, respectively), Cw, Rw- chest wall compliance and viscosity, Ps- respiratory muscles, Pw- intrapleural pressure, P- pressure in the mouth (if breathing is supported with CPAP, P = CPAP). Numbered boxes describe lobes (i = 1, 2 – left upper, lower lobe, 3, 4, 5 – right upper, middle, lower lobe): Rp_i _– the "resistance" of the bronchi that may collapse, Rv_i _– the resistance of smallest bronchi, Cc_i _– air compressibility, Ca_i _– the lobe compliance, Rt_i _– lobe tissue viscosity.

The investigations were conducted on the VRS built-up previously [1-4,6]. Fig. 1 presents its general structure. The VRS data for a healthy human being (the standard patient) have been collected on the basis of wide accessible literature.

**Figure 2:**
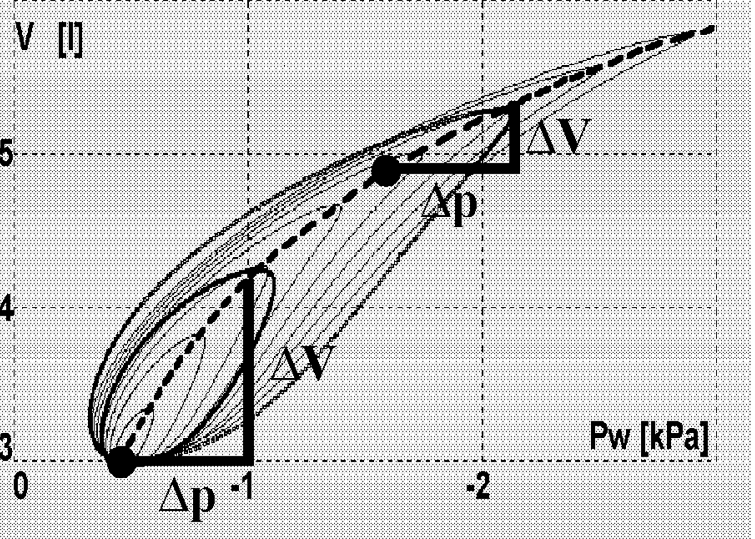
**Influence of the working point on the differential lungs compliance**. V – lungs volume, Pw – the difference between intrapleural and upper airways pressures, which is equal to the transpulmonary pressure when the air flow is equal to zero (thus, the broken line presents the nonlinear lungs compliance). The greater the lungs volume, the smaller the lungs volume increase (ΔV) caused by a particular pressure increase (Δp), *i.e*. the greater the volume, the smaller the differential compliance ΔV/Δp. Solid curves (hystereses) illustrate influence of Raw on the relationship between the volume and pressure during breathing with different deepness of breaths.

Nonlinearity of the respiratory system compliance (Fig. 2) and changeability of the nonlinear Airway Resistance (**Raw) **(Appendix – the formulas A.4,5) are those VRS features, which are especially important for this paper (see Appendix for other details). Taking into account physiological differences, Raw has been divided into:

(a) Resistances of the upper airways (the main bronchi, the trachea, *etc*.).

(b) Resistances that depend on lungs volume (Rv_i _in Fig. 1) – the resistance of smallest bronchi (*i.e*. of highest generations), which are component of the lung tissue, and thus their size depends on the current lungs volume.

(c) Resistances that depend on transmural pressure (Rp_i _in Fig. 1) – the resistance of bronchi of the middle generations, the diameter of which depends on the transmural pressure, i.e. on the difference between pressure inside the bronchi and the intrapleural pressure. Since both the intrapleural pressure and pressure inside the bronchi change during respiration, the bronchi diameter (thus also Rp_i_) changes, too. In particular, if the transmural pressure is negative during expiration, such bronchi collapse and the airflow does not depend on the pressure gradient. Thus, the simplified idea of "resistance" as the coefficient of proportionality between the flow Q and the pressure gradient Δp (Δp = Q•R) loses the sense. Therefore, the direct dependence between the airflow and the pressures is used in VRS, in fact (the way of the dependence description has been explained in [6], whereas the final formula is presented in Appendix). Nevertheless, the term "resistance" is used in the paper because of common practice.

In the text below, the "Raw = norm" means the airways of the standard person, *i.e*. the normal Raw. However, since changeable Rp_i _and Rv_i _are the most meaningful components of Raw, it is impossible to characterize this "normal resistance" with simple numeric value(s). Therefore, the "Raw = norm" means that the airflow depends on the pressures as for the standard person. Consequently, an obstruction described below with the "Raw = X·norm" means that the airflows are "X" times smaller than they would be for the standard person under the same instantaneous conditions. Since the analyzed obstruction concerns bronchi of the middle generation, resistance of which is described with the formula A.5 (see Appendix), Raw = X·norm means that the coefficient k in the formula A.5 is X times greater than for the standard person. In this paper, results for exemplary virtual patients, for which X = 1, 4, 8, and 16, have been analyzed. To connect this mathematical description with commonly used clinical classification, spirometry was performed [6]. According to European Respiratory Society and American Thoracic Society criteria, simulated disease with Raw = 4·norm should be classified as a moderate obstruction while with Raw = 8·norm and Raw = 16·norm as severe and very severe obstructions.

### 2.2. Experimental procedures

Investigation on VRS enables analysis of a problem with the "step by step" method, *i.e*. it is possible to change a single factor to observe results, then to change another one to observe further results, *etc*. Such an approach enables easier determination which one(s) from the set of initially postulated factors really influences an analyzed phenomenon. The "step by step" method is usually impossible in the case of living models because of feedback between all factors and parameters.

This paper regards the reason of efficacy of ventilatory support with CPAP in the obstructive disease, when the obstruction concerns the bronchi that depend on the transmural pressure, i.e. when Raw is increased because of increased Rp_i_. Thus, breathing improvement with CPAP was the analyzed phenomenon. A change of the tidal volume (V_T_) was treated as an index of the improvement. The values of Raw and/or the breathing frequency were changed to analyze the dependence of V_T _on CPAP. The other factors remained constant. Especially, the respiratory muscle effort during particular inspirations was unchanged.

Finally, influence of CPAP on minute ventilation for different breathing frequency was analyzed to find the optimal frequency and the CPAP value.

The muscle effort has to be well defined to be used in quantitative analysis, esp. in a computer model. Generally, oxygen consumption is commonly accepted as the best index of the effort of living being. However, such a chemical index cannot be used in simulations of mechanics, which is the matter of this paper. The mechanical work done could be a good index in the case of mechanical (physical) devices. Unfortunately, the physical work is proportional to the movement (*e.g*. the work is equal to the force times displacement or pressure times volume change) while there is no simple connection between the movement and the organism effort. Isovolumetric or isometric efforts can be examples. Moreover, such a work cannot be a presettable parameter of the simulations because it may be determined with integration of time-related pressures and volumes, *i.e*. it may be determined only after the simulation.

**Figure 3:**
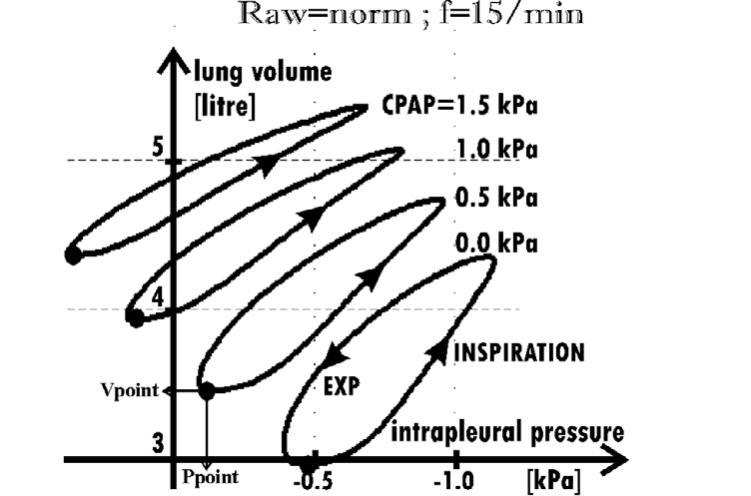
**Lung volume versus intrapleural pressure**. for Raw = norm, four values of CPAP, and the breathing frequency equal to 15/min (typical for healthy persons). Black points [Ppoint, Vpoint] determine the working points showed in Fig. 2 (where Pw = Ppoint-CPAP).

For the above reasons, the amplitude of the force developed by the respiratory muscles has been assumed as the effort index. In the VRS, the force is represented by the pressure caused by this force (Ps in Fig. 1). It can be proved that the intrapleural pressure change (ΔPw) caused by Ps can be determined with the following formula:

ΔPw=−Ps⋅CwdCad+Cwd
 MathType@MTEF@5@5@+=feaafiart1ev1aaatCvAUfKttLearuWrP9MDH5MBPbIqV92AaeXatLxBI9gBaebbnrfifHhDYfgasaacH8akY=wiFfYdH8Gipec8Eeeu0xXdbba9frFj0=OqFfea0dXdd9vqai=hGuQ8kuc9pgc9s8qqaq=dirpe0xb9q8qiLsFr0=vr0=vr0dc8meaabaqaciaacaGaaeqabaqabeGadaaakeaacqqHuoarcqWGqbaucqWG3bWDcqGH9aqpcqGHsislcqWGqbaucqWGZbWCcqGHflY1daWcaaqaaiabdoeadjabdEha3naaBaaaleaacqWGKbazaeqaaaGcbaGaem4qamKaemyyae2aaSbaaSqaaiabdsgaKbqabaGccqGHRaWkcqWGdbWqcqWG3bWDaaWaaSbaaSqaaiabdsgaKbqabaaaaa@446A@

where: Cw_d_, Ca_d _– differential compliances of the chest wall and lungs, respectively. Since Ca_d _falls with the lungs volume increase (Fig. 2, the formula A.3), the greater the volume, the greater the ΔPw value caused by the same Ps (hence changes of the intrapleural pressure in Fig. 3 are bigger for the greater CPAP despite unchanged Ps). However, although the increase in the intrapleural pressure rises, increase in the lungs volume falls because of the following dependence:

ΔV=Ps⋅Cwd1+Cwd/Cad
 MathType@MTEF@5@5@+=feaafiart1ev1aaatCvAUfKttLearuWrP9MDH5MBPbIqV92AaeXatLxBI9gBaebbnrfifHhDYfgasaacH8akY=wiFfYdH8Gipec8Eeeu0xXdbba9frFj0=OqFfea0dXdd9vqai=hGuQ8kuc9pgc9s8qqaq=dirpe0xb9q8qiLsFr0=vr0=vr0dc8meaabaqaciaacaGaaeqabaqabeGadaaakeaacqqHuoarcqWGwbGvcqGH9aqpcqWGqbaucqWGZbWCcqGHflY1daWcaaqaaiabdoeadjabdEha3naaBaaaleaacqWGKbazaeqaaaGcbaGaeGymaeJaey4kaSIaem4qamKaem4DaC3aaSbaaSqaaiabdsgaKbqabaGccqGGVaWlcqWGdbWqcqWGHbqyaaWaaSbaaSqaaiabdsgaKbqabaaaaa@43E8@

## 3. Results

### 3.1. CPAP and respiratory system nonlinearity

The value of Raw strongly depends on the bronchi diameter (according to the Poiseuille's law for the ideal tube, the resistance is inversely proportional to the fourth power of the radius). Therefore, since the diameter depends on the transmural pressure (the difference between pressures inside and outside the bronchus), CPAP influences Raw: the greater the CPAP value, the smaller the Raw value. Hence it appears that CPAP may make breathing easier because a smaller part of the force developed by the respiratory muscles has to be used against Raw (which is illustrated by narrow hystereses for the greater CPAP in Fig. 3).

On the other hand, however, CPAP may make breathing harder. Indeed, if the airflow is equal to zero, as at the inspiration or expiration end, then the pressure inside the lungs is equal to the pressure inside the airways. In particular, the pressure inside the lungs at the inspiration beginning is equal to CPAP, which means that the starting lungs volume is greater than would be for the pressure equal to zero. Since the lungs compliance is nonlinear (see the formula A.3), CPAP moves the working point towards the smaller differential compliance (Fig. 2). As the consequence, the same force developed by the respiratory muscles causes smaller V_T _(the formula 2). Therefore, the greater the CPAP value, the more horizontal the hystereses as shown in Fig. 3.

**Figure 4:**
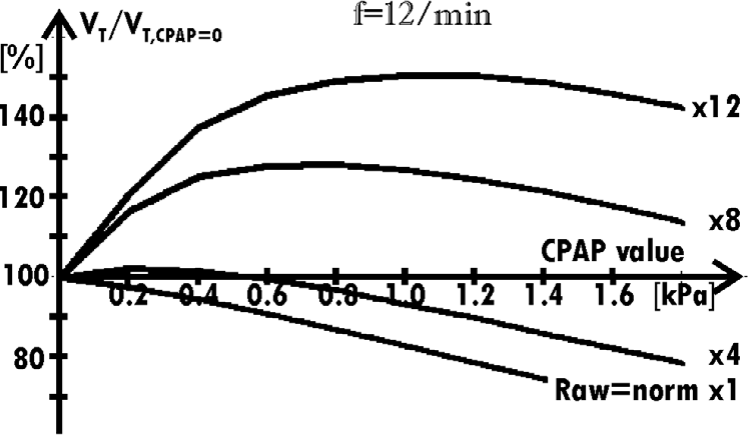
**Dependence of the relative value of V_T _on the CPAP value**. for the typical breathing frequency and four values of the airway resistance (Raw = norm, 4·norm, 8·norm and 12·norm).

The above analysis suggests that CPAP may either improve or worsen the breathing. Simulations showed that the result of the CPAP use depends on the Raw value. In the case of healthy persons, Raw appears small enough to make breathing efficient, and thus additional decreasing of Raw has not significant influence on lungs ventilation. Therefore, CPAP decreases V_T _because negative influence of the decreased compliance dominates over such an unnecessary resistance decrease (Fig. 4 – the results for Raw = norm and Raw = 4·norm).

In the case of the significantly increased Raw, the use of CPAP gives contrary result: CPAP increases V_T _(Fig. 4 – the results for Raw = 8·norm and Raw = 12·norm), and thus CPAP makes breathing easier. As it will be shown below, a relatively small fall of V_T _caused by a differential compliance decrease is compensated with a surplus by the fact that the lungs can empty themselves through the Raw decreased by CPAP.

### 3.2. CPAP and breathing frequency

**Figure 5:**
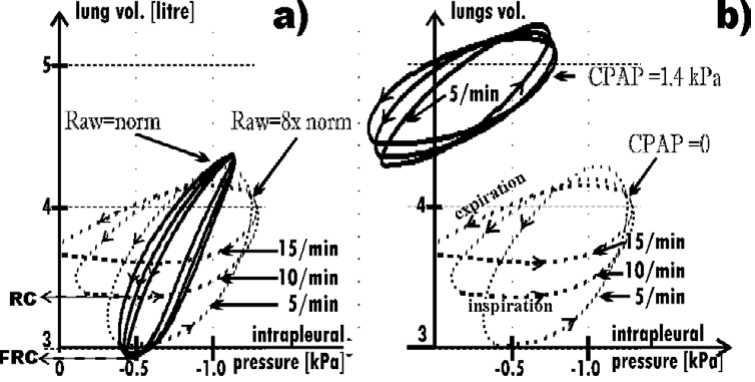
**Sets of hystereses (lung volume versus intrapleural pressure) for three breathing frequencies**. **a) **The normal (solid curves) and the increased (dotted curves) Raw; FRC – functional residual capacity, RC – residual capacity, which is greater than FRC in the case of the increased Raw because the lungs have too small time to empty themselves to the FRC level through the increased Raw. Therefore, if Raw is increased, then V_T _depends on the breathing frequency. Note that the end-inspiratory volume is almost independent from the frequency. **b) **Raw = 8·norm, without (dotted curves) and with the support (solid curves).

As shown in Fig. 5a, if Raw = norm then V_T _does not depend on the breathing frequency (solid lines). However, when Raw is high, V_T _depends on the frequency significantly (broken lines). If Raw is increased, V_T _is small for the normal breathing frequency (≈12–15/min) because lungs have too short time to empty themselves through the increased Raw. Therefore, the minimal lungs volume, *i.e*. the residual capacity (RC), is about 0.7 liter greater than the functional residual capacity (FRC) in the simulation for Raw = 8·norm and the frequency equal to 15/min (Fig. 5a). V_T _attains the normal value not sooner than the breathing frequency falls significantly. Note that such a frequency decrease is commonly known, as it is a natural defense mechanism in the case of obstructive lung disease [7]. However, if the frequency decreases ***n ***times, breath deepness should increase ***n ***times to remain the minute ventilation on the required level. Unfortunately, as it will be indicated in the Discussion, deep breaths are much more energy-consuming than frequent but shallow breathing.

**Figure 6:**
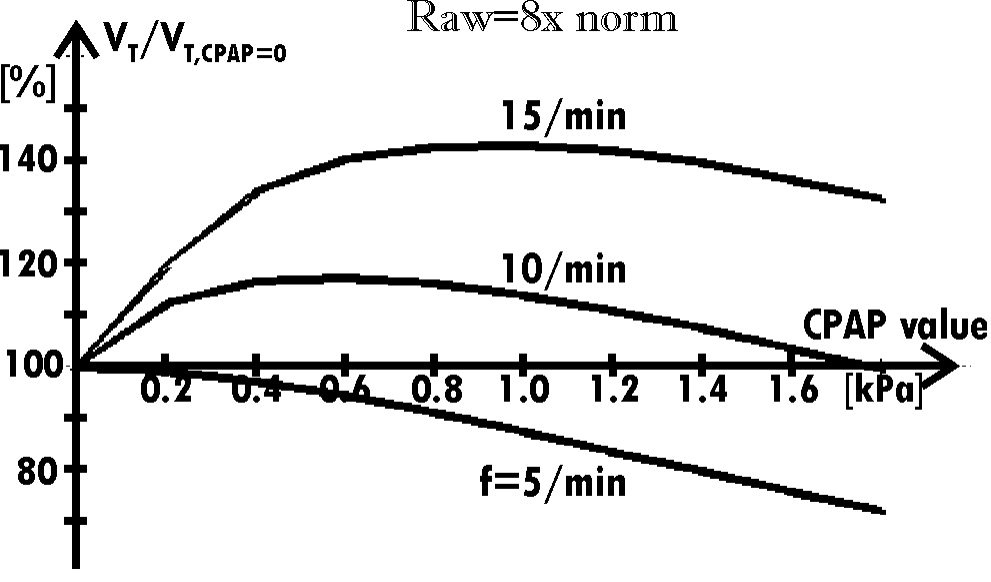
**Dependence of the relative value of V_T _on the CPAP value**. for the increased Raw, for three breathing frequencies.

CPAP reduces the influence of the frequency on V_T _(solid lines in Fig. 5b) because it increases V_T _in the case of higher frequencies while it decreases V_T _for lower frequencies. Indeed, if breathing frequency is higher, lungs need less time to empty themselves through the Raw decreased by CPAP. For that reason, CPAP increases V_T_. On the other hand, if breathing frequency was low, lungs would have enough time to empty themselves without the CPAP support. Hence it appears that CPAP is not helpful in this case. In fact, it appears unprofitable since it decreases V_T _because of a shift of the working point toward the smaller lungs compliance (as in the case of the normal Raw – compare the curve for Raw = norm in Fig. 4 with the curve for f = 5/min in Fig. 6).

Summarizing: in the obstructive lung disease, CPAP improves breathing with the normal frequency but it is unprofitable in the case of breathing with low frequency (Fig. 6).

**Figure 7:**
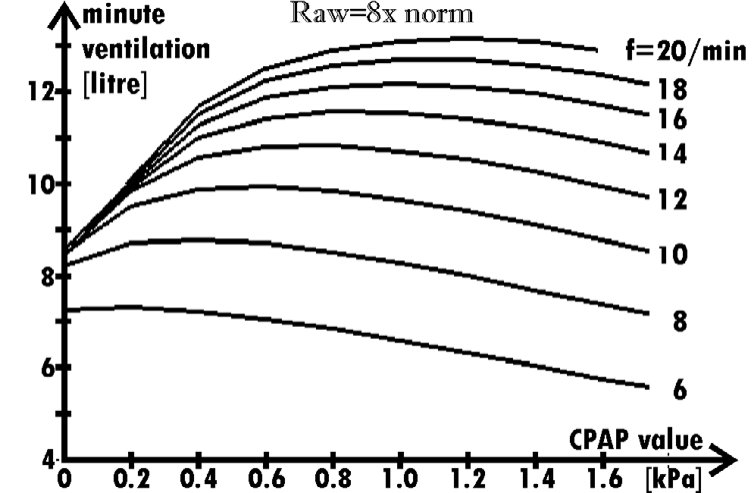
**Dependence of the minute ventilation on the CPAP value for several breathing frequencies when Raw = 8norm**. (the force developed by the respiratory muscles during particular inspirations is the same for all the frequencies). Note that the ventilation cannot be increased by means of the frequency rise if the respiration is not supported with CPAP.

Since the minute ventilation determines blood oxygenation, it is a respiration parameter more important than V_T_. Therefore, its dependence on CPAP for different frequencies was analyzed, too. Fig. 7 presents the results. Note that if a patient suffering from the analyzed obstructive disease is not supported with CPAP, there is a limit of minute ventilation increase with the frequency rise (the limit is equal to 8.5 liter/min in the case of the exemplary virtual patient, which results are shown in Fig. 7). Existence of such limit seems to be the most interesting result.

## 5. Discussion

As Fig. 5 shows, the maximal (*i.e*. end-inspiratory) volume is almost independent from the breathing frequency. Therefore, the frequency influence on V_T _is mainly caused by its influence on RC. Hence it appears that expiration rather than inspiration is critical in the analyzed obstructive lungs disease. However, although expiration is critical directly, its meaning is connected with its influence on inspiration, esp. its influence on the starting volume (*i.e*. RC), which determines the working point. For that reason, inspiration, especially the inspiratory work, has to be analyzed, too. In particular, such frequency and CPAP, for which the work is minimal, may be treated as the optimal ones.

The real work of the respiratory system (measured with oxygen consumption) and its mechanical work **W **(described with the formula dW = p·dV, where: p-pressure, V-volume) are not exactly the same (especially in the case of isovolumetric processes). Nevertheless, changes of the mechanical work may usually estimate changes of the real work.

Let us assume initially, that both Raw and the respiratory system compliance **C **are constant (the system is linear) and a patient needs ventilation of **V **liters per the time unit **T**.

If such ventilation is carried out by one deep breath, the work per **T **against lungs elasticity will be as follows:

W1=1C⋅V22~Ps2
 MathType@MTEF@5@5@+=feaafiart1ev1aaatCvAUfKttLearuWrP9MDH5MBPbIqV92AaeXatLxBI9gBamXvP5wqSXMqHnxAJn0BKvguHDwzZbqegyvzYrwyUfgarqqtubsr4rNCHbGeaGqiA8vkIkVAFgIELiFeLkFeLk=iY=Hhbbf9v8qqaqFr0xc9pk0xbba9q8WqFfeaY=biLkVcLq=JHqVepeea0=as0db9vqpepesP0xe9Fve9Fve9GapdbaqaaeGacaGaaiaabeqaamqadiabaaGcbaGaem4vaC1aaSbaaSqaaiabigdaXaqabaGccqGH9aqpdaWcaaqaaiabigdaXaqaaiabdoeadbaacqGHflY1daWcaaqaaiabdAfawnaaCaaaleqabaGaeGOmaidaaaGcbaGaeGOmaidaaiabc6ha+jabbcfaqnaaDaaaleaacqqGZbWCaeaacqaIYaGmaaaaaa@4D3E@

If the ventilation is carried out by two breaths of two times smaller deepness, the work will be:

W2=2⋅1C⋅(V/2)22=12⋅(1C⋅V22)
 MathType@MTEF@5@5@+=feaafiart1ev1aaatCvAUfKttLearuWrP9MDH5MBPbIqV92AaeXatLxBI9gBaebbnrfifHhDYfgasaacH8akY=wiFfYdH8Gipec8Eeeu0xXdbba9frFj0=OqFfea0dXdd9vqai=hGuQ8kuc9pgc9s8qqaq=dirpe0xb9q8qiLsFr0=vr0=vr0dc8meaabaqaciaacaGaaeqabaqabeGadaaakeaacqWGxbWvdaWgaaWcbaGaeGOmaidabeaakiabg2da9iabikdaYiabgwSixpaalaaabaGaeGymaedabaGaem4qameaaiabgwSixpaalaaabaWaaeWaaeaacqWGwbGvcqGGVaWlcqaIYaGmaiaawIcacaGLPaaadaahaaWcbeqaaiabikdaYaaaaOqaaiabikdaYaaacqGH9aqpdaWcaaqaaiabigdaXaqaaiabikdaYaaacqGHflY1daqadaqaamaalaaabaGaeGymaedabaGaem4qameaaiabgwSixpaalaaabaGaemOvay1aaWbaaSqabeaacqaIYaGmaaaakeaacqaIYaGmaaaacaGLOaGaayzkaaaaaa@4CEB@

Thus, since W2=12⋅W1
 MathType@MTEF@5@5@+=feaafiart1ev1aaatCvAUfKttLearuWrP9MDH5MBPbIqV92AaeXatLxBI9gBaebbnrfifHhDYfgasaacH8akY=wiFfYdH8Gipec8Eeeu0xXdbba9frFj0=OqFfea0dXdd9vqai=hGuQ8kuc9pgc9s8qqaq=dirpe0xb9q8qiLsFr0=vr0=vr0dc8meaabaqaciaacaGaaeqabaqabeGadaaakeaacqWGxbWvdaWgaaWcbaGaeGOmaidabeaakiabg2da9maalaaabaGaeGymaedabaGaeGOmaidaaiabgwSixlabdEfaxnaaBaaaleaacqaIXaqmaeqaaaaa@36A0@, the more shallow and frequent breathing is less energy-consuming. Since the mean airflow is approximately the same in the both cases (it is equal to V/T), the work against Raw is approximately the same, too (it has to be stressed that Raw during inspiration is smaller than during expiration – see the formula A.5). Hence it appears that differences in the work against the elasticity are significant.

The above consideration shows that deep breathing with low frequency is more energy-consuming than more frequent breathing. Since the compliance decreases according to lung volume rise, deep breaths are much more energy-consuming, in fact. Hence it appears that since breathing is one of the most energy-consuming physiological processes, the organism should breathe as frequently and shallowly as it is possible under actual conditions (certainly, since V_T _has to be greater than the dead space to ventilate alveoli, the breath deepness cannot be too small). To determine the optimal frequency for a particular CPAP value, the inspiratory work against the lungs compliance for constant minute ventilation was approximated for the different frequencies. It was assumed that: (a) the work is proportional to squared value of Ps (as in the formula 3), and (b) the lungs compliance depends on the working point but it does not depend on the breath deepness.

**Figure 8:**
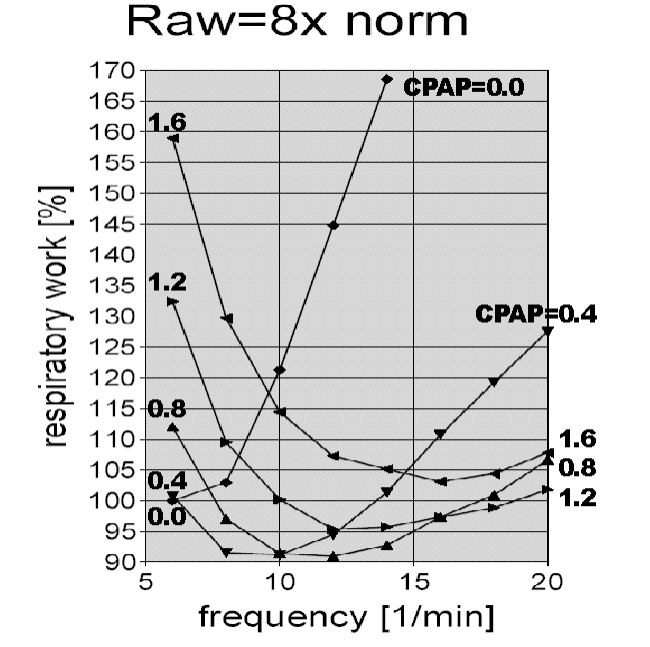
**Breathing frequency versus an approximation of the respiratory work against lungs compliance**. for the minute ventilation equal to 8 l/min and CPAP = 0.0, 0.4, 0.8, 1.2, and 1.6 kPa (the respiratory work is presented as the percentage of the work when CPAP = 0 and the frequency = 6/min). CPAP influences optimal frequency, *i.e*. the frequency, for which the respiratory work is minimal: the greater the CPAP value, the greater the optimal frequency.

Fig. 8 shows results for the recalculated data presented in Fig. 7 (Ps, for a particular frequency and CPAP value, was changed to obtain the minute ventilation equal to 8 l/min). As Fig. 8 shows, the optimal frequency depends on the CPAP value. The results suggest that if Raw = 8·norm then the optimal CPAP value is equal to 0.6 kPa; the optimal frequency for such CPAP value is equal to 10/min. It has to be stressed that the minima in Fig. 8 should be more significant, in fact, since: (a) the real inspiratory work for low frequencies is greater because the compliance depends on the breath deepness, and (b) the minute ventilation in the case of higher frequencies has to be greater than 8 l/min to keep the alveolar ventilation unchanged.

It is commonly known that patients suffering from lung obstructive disease breathe with low frequency. Although it is breathing in the energy-consuming way, they have no choice. As the simulations showed (Fig. 7), if there is no support with CPAP (*i.e*. CPAP = 0), rise of the frequency (above 8/min for Raw = 8·norm) is pointless because such a rise does not increase the minute ventilation. That seems to be the true reason of the fact that breathing with low frequency is the well-known natural defense mechanism in the case of patients suffering from the obstructive lungs disease. Our explanation, which bases on the simulations and stresses problems during the expiration, is different than the commonly accepted but rather intuitive explanation, which stresses the influence of the work against Raw during the inspiration [7]. If CPAP is used, the above natural defense mechanism is not necessary.

## 6. Conclusion

1. It is commonly accepted now that a breathing frequency decrease in obstructive lungs disease is connected with an increase of the inspiratory work against Raw. Our simulations, however, suggest that problems of the expiration rather than the inspiration are the main cause of such frequency fall. The simulations indicated that the minute ventilation rises with the breathing frequency increase only if the frequency is smaller than certain boundary value because of difficulties with lungs emptying during too short expiration. The emptying through the increased Raw needs more time than through the normal Raw, which causes that in the severe obstructive disease the boundary value is smaller than the normal breathing frequency. Therefore, a patient has to breathe slowly since more frequent breathing would not increase the ventilation but it would increase the minute inspiratory work.

2. The simulations suggest that the CPAP efficacy is connected mainly with possibility of effective breathing with the frequency, which is close to the normal one. Therefore, CPAP seems to be helpful only when Raw is increased significantly, *i.e*. when the frequency that is economic without the CPAP support is smaller than the normal one.

3. CPAP makes breathing easier because it decreases required inspiratory work against the reduced Raw (which is commonly known) as well as the lungs compliance since: (a) the work against the compliance is approximately proportional to the squared tidal volume, (b) the tidal volume is approximately inversely proportional to the breathing frequency, and (c) CPAP enables more frequent breathing.

4. The optimal breathing frequency depends on both the CPAP value and the obstruction level.

## Appendix

### Model description

Fig. 1 presents the general structure of the computer model that is postulated to be VRS. The main features of the model are:

1) nonlinearity of the meaningful model elements;

2) separation of the lungs and chest;

3) division of the lungs into lobes (i = 1...5 identifies the upper left lobe ....the bottom right lobe, respectively);

4) division of the airway resistance into:

(a) resistances that depend on the lung volume (Rv_i _in Fig. 1) – resistances of the smallest bronchi that are a component of the lung tissue;

(b) resistances that depend on the transmural pressure (Rp_i _in Fig. 1) – resistances of the bronchi that may collapse; and

(c) resistances of the large bronchi;

5) many others such as influence of gravity (G_i_), air compressibility (Cc_i_), *etc*.

Data for the model for the "standard" (healthy) human being have been collected on the basis of accessible literature. Some of the model elements in Fig. 1 represent parameters, which are described by single numbers. Such parameters are: Ru, R, RL, Rr, Rti, Rw, and L, LL, Lr – (resistances of mouth, trachea, left and right main bronchi, lung tissue, chest tissue, and inertance of trachea, left and right main bronchi, respectively). R, R_L_, R_r_, L, L_L_, L_r _were calculated from dimensions of trachea or main bronchi. Rt_i _and Rw were estimated on the basis of data from [8].

Nonlinear model elements are described in the following way:

1. The air compressibility 'compliance' symbolized by Cc_i _in Fig. 1, *i.e*. the dependence between the alveolar pressure (Patm+Pa_i_), the lobe volume Va_i_, and the volume (Va_i_+Vc_i_) that the air from the lobe would occupy at the atmospheric pressure (Patm):

Pa_i _= Patm·Vc_i_/Va_i_

2. Dependence between the trans-wall pressure and the chest volume (*i.e*. the chest wall compliance symbolized by Cw in Fig. 1) is described with the formula:

ptw=C1⋅Vw−C2Vw−C3
 MathType@MTEF@5@5@+=feaafiart1ev1aaatCvAUfKttLearuWrP9MDH5MBPbIqV92AaeXatLxBI9gBaebbnrfifHhDYfgasaacH8akY=wiFfYdH8Gipec8Eeeu0xXdbba9frFj0=OqFfea0dXdd9vqai=hGuQ8kuc9pgc9s8qqaq=dirpe0xb9q8qiLsFr0=vr0=vr0dc8meaabaqaciaacaGaaeqabaqabeGadaaakeaacqWGWbaCdaWgaaWcbaGaemiDaqNaem4DaChabeaakiabg2da9iabdoeadjabigdaXiabgwSixlabdAfawnaaBaaaleaacqWG3bWDaeqaaOGaeyOeI0YaaSaaaeaacqWGdbWqcqaIYaGmaeaadaGcaaqaaiabdAfawnaaBaaaleaacqWG3bWDaeqaaOGaeyOeI0Iaem4qamKaeG4mamdaleqaaaaaaaa@424F@

where: Ptw – the trans-wall pressure, Vw – the chest volume, coefficients C1, C2, and C3 depend on simulated patient (C1 = 0.3 kPa/l, C2 = 1.722 kPa·l^1/2^, C3 = 1.5 l for the standard patient). The first part of the formula represents differential linearity of the chest-wall compliance for greater volumes, whereas the second part describes the limit of chest compression.

3. Dependence between the trans-pulmonary pressure and the lobe volume (*i.e*. the lobe compliance symbolized by Ca_i _in Fig. 1) is described with the formula:

Ptpsi=1[kPa]⋅exp⁡(C4+Vai⋅C5ui)
 MathType@MTEF@5@5@+=feaafiart1ev1aaatCvAUfKttLearuWrP9MDH5MBPbIqV92AaeXatLxBI9gBaebbnrfifHhDYfgasaacH8akY=wiFfYdH8Gipec8Eeeu0xXdbba9frFj0=OqFfea0dXdd9vqai=hGuQ8kuc9pgc9s8qqaq=dirpe0xb9q8qiLsFr0=vr0=vr0dc8meaabaqaciaacaGaaeqabaqabeGadaaakeaacqWGqbaucqWG0baDcqWGWbaCcqWGZbWCdaWgaaWcbaGaemyAaKgabeaakiabg2da9iabigdaXmaadmaabaGaem4AaSMaemiuaaLaemyyaegacaGLBbGaayzxaaGaeyyXICTagiyzauMaeiiEaGNaeiiCaa3aaeWaaeaacqWGdbWqcqaI0aancqGHRaWkcqWGwbGvcqWGHbqydaWgaaWcbaGaemyAaKgabeaakiabgwSixpaalaaabaGaem4qamKaeGynaudabaGaemyDau3aaSbaaSqaaiabdMgaPbqabaaaaaGccaGLOaGaayzkaaaaaa@51CE@

where: Ptps_i _– the static trans-pulmonary (recoil) pressure concerning the lobe of number i, Va_i _– the volume of this lobe, C4 and C5 depend on simulated patient (C4 = -2.76, C5 = 0.66 l^-1 ^for all i, for the standard homogenous lungs), u_i _– coefficient determining what part of the whole lung is this particular lobe (u = [0.2, 0.25, 0.15, 0.2, 0.2]). Note that Va_i_·C5/u_i _= V·u_i_·C5/u_i _= V·C5 for all i (where V- the total lungs volume). It means that it has been assumed initially that each lobe of the homogenous lungs has the same elastic properties as the whole lung.

The formulas (A.2) and (A.3) have been chosen because they well approximate characteristic dependence of the pressures on the volumes. Numeric values of the parameters C1, ..., C5 fit the graphic dependence presented in [9,10].

4. The resistance Rv_i _that depends on the lobe volume Va_i _(a_RVi _– a proportionality coefficient, ccVa_i _– critical closing volume) is described with the formula:

Rv_i _= a_RVi_/(Va_i_-ccVa_i_)

5. The resistance symbolized by Rp_i _in Fig. 1, *i.e*. the dependence of the airflow ***f ***on: the pleural pressure Pp, the pressure in the main bronchi Pb, and the pressure in the lobe Pa_i _(b_i _and k_i _– coefficients dependent on the simulated patient; for the standard patient b_i _= 0.4 kPa, k_i _= u_i· _0.063 kPa·sec/l) is described with the formula:

f=Pb−Pai−bi⋅arctg(bi⋅Pb−Paibi2+(Pb−Pp)⋅(Pai−Pp))ki
 MathType@MTEF@5@5@+=feaafiart1ev1aaatCvAUfKttLearuWrP9MDH5MBPbIqV92AaeXatLxBI9gBamXvP5wqSXMqHnxAJn0BKvguHDwzZbqegyvzYrwyUfgarqqtubsr4rNCHbGeaGqiA8vkIkVAFgIELiFeLkFeLk=iY=Hhbbf9v8qqaqFr0xc9pk0xbba9q8WqFfeaY=biLkVcLq=JHqVepeea0=as0db9vqpepesP0xe9Fve9Fve9GapdbaqaaeGacaGaaiaabeqaamqadiabaaGcbaGaemOzayMaeyypa0ZaaSaaaeaacqWGqbaucqWGIbGycqGHsislcqWGqbaucqWGHbqydaWgaaWcbaGaemyAaKgabeaakiabgkHiTiabdkgaInaaBaaaleaacqWGPbqAaeqaaOGaeyyXICTaemyyaeMaemOCaiNaem4yamMaemiDaqNaem4zaC2aaeWaaeaacqWGIbGydaWgaaWcbaGaemyAaKgabeaakiabgwSixpaalaaabaGaemiuaaLaemOyaiMaeyOeI0IaemiuaaLaemyyae2aaSbaaSqaaiabdMgaPbqabaaakeaacqWGIbGydaqhaaWcbaGaemyAaKgabaGaeGOmaidaaOGaey4kaSYaaeWaaeaacqWGqbaucqWGIbGycqGHsislcqWGqbaucqWGWbaCaiaawIcacaGLPaaacqGHflY1daqadaqaaiabdcfaqjabdggaHnaaBaaaleaacqWGPbqAaeqaaOGaeyOeI0IaemiuaaLaemiCaahacaGLOaGaayzkaaaaaaGaayjkaiaawMcaaaqaaiabdUgaRnaaBaaaleaacqWGPbqAaeqaaaaaaaa@7C39@

The formula (A.5) is the solution of the nonlinear differential equation (A.5a):

−dP=f⋅kX⋅P2+b2P2⋅dx
 MathType@MTEF@5@5@+=feaafiart1ev1aaatCvAUfKttLearuWrP9MDH5MBPbIqV92AaeXatLxBI9gBaebbnrfifHhDYfgasaacH8akY=wiFfYdH8Gipec8Eeeu0xXdbba9frFj0=OqFfea0dXdd9vqai=hGuQ8kuc9pgc9s8qqaq=dirpe0xb9q8qiLsFr0=vr0=vr0dc8meaabaqaciaacaGaaeqabaqabeGadaaakeaacqGHsislcqWGKbazcqWGqbaucqGH9aqpcqWGMbGzcqGHflY1daWcaaqaaiabdUgaRbqaaiabdIfaybaacqGHflY1daWcaaqaaiabdcfaqnaaCaaaleqabaGaeGOmaidaaOGaey4kaSIaemOyai2aaWbaaSqabeaacqaIYaGmaaaakeaacqWGqbaudaahaaWcbeqaaiabikdaYaaaaaGccqGHflY1cqWGKbazcqWG4baEaaa@46CA@

Details, esp. the derivation of the formula (as well as explanations of other formulas and model verification) has been presented precisely in [6].

Coefficients b_i _determine how quickly the resistance falls with transmural pressure increase (the smaller b_i_, the quicker the resistance fall). k_i _describe the resistance for the infinite (theoretically) transmural pressure. Thus, b_i _are approximately connected with mechanical bronchi wall properties, while k_i _reflect bronchi internal dimensions. Such mathematical description of Rp_i _enables to simulate – with one formula – both the airflow auto-limitation employed in spirometry and the commonly known, experimental dependence of the airway resistance on lungs volume.

If a disease is examined, appropriate model parameters are adequately changed to simulate this disease. For example, increase of C4 simulates restrictive lung disease connected with the lung compliance fall, whereas increase of C5 may simulate restrictive lung disease because of the lung (lobe) capacity decrease.

## Authors' contributions

TG conceived the study, participated in the study design, performed physiological, physical, and mathematical analysis, wrote computer programs, and drafted the manuscript. MD participated in the study design, simulations, and results analysis. Both authors read and approved the final manuscript.
